# Wide Bandpass and Narrow Bandstop Microstrip Filters Based on Hilbert Fractal Geometry: Design and Simulation Results

**DOI:** 10.1371/journal.pone.0115412

**Published:** 2014-12-23

**Authors:** Yaqeen S. Mezaal, Halil T. Eyyuboglu, Jawad K. Ali

**Affiliations:** 1 Electronic and Communication Engineering Department, Cankaya University, Ankara, Turkey; 2 Microwave Research Group, Electrical Engineering Department, University of Technology, Baghdad, Iraq; Northwestern Polytechnical University, China

## Abstract

This paper presents new Wide Bandpass Filter (WBPF) and Narrow Bandstop Filter (NBSF) incorporating two microstrip resonators, each resonator is based on 2^nd^ iteration of Hilbert fractal geometry. The type of filter as pass or reject band has been adjusted by coupling gap parameter (d) between Hilbert resonators using a substrate with a dielectric constant of 10.8 and a thickness of 1.27 mm. Numerical simulation results as well as a parametric study of d parameter on filter type and frequency responses are presented and studied. WBPF has designed at resonant frequencies of 2 and 2.2 GHz with a bandwidth of 0.52 GHz, −28 dB return loss and −0.125 dB insertion loss while NBSF has designed for electrical specifications of 2.37 GHz center frequency, 20 MHz rejection bandwidth, −0.1873 dB return loss and 13.746 dB insertion loss. The proposed technique offers a new alternative to construct low-cost high-performance filter devices, suitable for a wide range of wireless communication systems.

## Introduction

The fractal term which indicates broken or fragmented parts was invented less than thirty years ago by one of history's most innovative mathematicians, Benoit Mandelbrot, in his pioneer work, The Fractal Geometry of Nature. Mandelbrot explained that many fractals are found in the nature that they could precisely form certain irregularly shaped objects or spatially non standardized phenomena in nature that cannot be attributed to Euclidean geometry, such as mountains or blood vessels. This means that fractals are in use with non-integer dimension. By expanding the idea of a fractional dimension, he concluded the term of fractal. He also described fractal as an irregular or fragmented geometric structure that can be divided into parts: each of which is (or approximately) a smaller-size copy of the whole. Mathematically, fractals are a kind of composite geometric shapes regularly display the property of self similarity, such that a small segment of it can be reduced as a fractional scale replica of the whole [1].

Fractals may be either random or deterministic. All obtainable fractal objects in nature are random in that they have been fashioned arbitrarily from non determined steps. Fractals that have been generated as a result of an iterative procedure, produced by consecutive dilations and conversions of a primary set, are deterministic. The fundamental fractal curves can be classified into six categories; these are Cantor, Koch, Minkowski, Hilbert, Sierpinski and Peano fractal geometries. All have the benefits of smallness and excellent quality performance. These properties attribute to fractal's two basic properties: self-similarity and space-filling. Self-similarity stands for a piece of the fractal geometry seems to be like that of the total structure for all time while the space-filling property means a fractal outline can be packed in a limited region as the iteration increases without increasing the whole area. The conventional fractals that generated by definite mathematic techniques always have exact self-similarity which can be known as well-regulated fractals. At present, fractal theory has been applied in many scientific research domains, and certainly turns on huge interests of microwave engineering researchers for designing latest microwave circuits and enhancing their performance in addition to miniaturization. However, this relevance rather dominantly focuses on antennas design as compared with other microwave circuit design including filters. Fractal structures can vary the current distribution of filter, and make it distributes along the conductor surface as opposed to the original simple patch surfaces, so the electric length will be increased [2], [3].

In this respect, fractals are going toward the design of a new generation of compact RF and microwave passive networks for wireless devices. Any wireless system relies on what is called the RF front-end stage which includes antennas, filters and diplexers, along with other passive elements such as capacitors, inductors and resistors. There is no problem whether the system is as influential as a cellular base-station, as sensitive as a super conducting satellite receiver or as small as a system-on-chip wireless device, the compactness and integration of such a front-end becomes always a key issue in terms of performance, robustness, packaging and cost. Fractal technology has been already applied in the miniaturization of another essential part of the wireless front-end. Compact fractal antennas for handsets, PDAs, cellular base-stations and high-speed data applications have been used in every small corner of the wireless world. The size compression and multiband qualifications of fractals allow well-organized, broadband and multi-purpose devices to be packed in places that were at length unreachable due to size, weight, or appearance constraints. Based on an analogous principle to filter and antenna miniaturization capabilities, fractal technology has been recently proven to become the most efficient way in packaging RF and microwave networks as well [4], [5].

On the other hand, microstrip bandpass and bandstop filters have developed rapidly and have led to spectacular demands for lower cost products with compact sizes and strong communication capabilities. In a microwave communication system, the bandpass filter (BPF) and bandstop filter (BSF) are essential components that are typically adopted in the transmitter and receiver system [6], [7]. One of the pioneers in the use of Hilbert fractal filter designs is Barra [8]. His work is focused on miniaturized superconducting filters using resonators based on Hilbert and Minkowski fractal layouts. He explored the miniaturization levels achievable by these resonators, with emphasize on the parameters which allow obtaining a good trade-off between compact size and losses. Several prototypes of four pole filters, with Chebychev and quasi elliptic responses, have been designed and fabricated. Microstrip lowpass filter operating within L-band application has been employed by making a slot in the ground plane in the form of Hilbert curve using defected ground structure (DGS) method as in [9]. The DGS structure has a flat lowpass characteristic and a sharp band-gap property compared to the conventional dumbbell DGS. In order to enhance the out-band suppression, an improved Hilbert fractal curve ring DGS cell model loaded with open-stubs was proposed. Based on the improved model, a compact L-band microstrip low-pass filter with periodic DGS was designed and studied. Typical and simplified cross-coupled spiral resonators with Hilbert configurations have been stated in [10] for a large coupling coefficient with comparison between each other. All of designs introduced in [10] have low insertion loss, high out-of-band rejection level and wider band frequency responses. Moreover, surface current distributions simulated by IE3D EM software package have been used to analyze the coupling regions in spiral and Hilbert configurations.

Narrow band dual loosely coupled resonator microstrip bandpass filters based on Hilbert fractal geometry with coupling stubs have been proposed for wireless application as in [11] within ISM band at fundamental frequency of 2.4 GHz. The proposed filter design topology is based on a single-mode microstrip resonators constructed from 2^nd^ and 3^rd^ iteration levels of Hilbert fractal geometry. The performance of each of the proposed filters has been analyzed using a method of moments (MoM) based software package, Microwave Office 2007, from Advanced Wave Research Inc. The new filters have small sizes and low insertion loss as well as high performances, which are very essential features in microstrip filter design theory. Bandstop filter using Hilbert defected ground structure (HDGS) has been built up and optimized using the fuzzy genetic algorithm as in [12]. This filter has been designed at 2.4 GHz center frequency and flat pass-band characteristics. The simulation results showed that this method has faster convergence rate than the traditional genetic algorithm. A second order bandpass filter is designed using DGS technique for wireless communication system has been reported in [13]. This filter has been constructed from Hilbert resonator which is etched on the bottom metal layer of the microstrip. The resonant characteristic of the fractal shaped DGS resonator has been analyzed. The effect of couplings between DGS resonators and input/output ports are also predicted. In [14], a study on the design of compact substrate integrated waveguide unit cell using Hilbert fractal slots has been introduced. It has been found that the suggested configuration offers a passband which is well below the cut-off frequency of the substrate integrated waveguide and hence, can be adopted in the design of miniaturized filter. Different orientations of the Hilbert curve are investigated and an optimal orientation that gives the best passband response has been extracted.

A Peano shaped dual-mode resonator has been presented to design a compact size microstrip bandpass filter with a quasi-elliptic response at 2.45 GHz [15]. The dual-mode ring resonator is composed of four sections, each with a structure based on the second iteration of Peano fractal geometry. This filter has narrow band frequency response with good electrical specifications. On the other hand, microstrip bandpass filters based on 3^rd^ iteration of Peano fractal resonators have been designed with and without tuning stubs as stated in [16]. The performance of these filters has been evaluated using IE3D EM software package. It has been found that adding a stub to each resonator provides the designer with an ability to tune the resulting filter response to the specified design frequency as well as 2^nd^ harmonic suppression in out of band region. Results show that these fractal filters possess a progressive size reduction with reasonable return loss and transmission responses. A modern narrow band bandpass filter based on Hilbert-zz fractal curve has been reported in [17]. This filter has more compactness as compared with the traditional Hilbert filter. Simulation results using Sonnet EM simulator show that the modeled filter has satisfactory return loss and transmission responses as well as blocked harmonics in out of band regions. More recently, new designs of microstrip bandpass filters, based on Hilbert fractal curve combined with SIR property have been presented and evaluated by using AWR2009 EM simulator as reported in [18]. The proposed fractal bandpass filters have been found to possess very compact sizes with good return loss and transmission responses as well as 2^nd^ order harmonic suppressions. In fact, very little attention has been paid to Hilbert bandstop filters as compared to Hilbert bandpass filters with two-dimensional (2-D) fractal curves as it can be seen from previous research work reported in the literature.

In this paper, new designs of Wide Bandpass Filter (WBPF) and Narrow Bandstop Filter (NBSF) based on Hilbert fractal resonators have been investigated using Sonnet EM simulator. The frequency responses of the proposed filters have been studied to observe the corresponding broad bandpass and narrow bandstop behaviors at a frequency around 2 GHz. Moreover, the phase dispersion and surface current densities on the surfaces of the proposed filters have been presented and analyzed. The proposed fractal filters have been found to possess compact sizes with flexible designs in addition to good frequency responses.

## Hilbert Fractal Geometry

**Figure 1:**
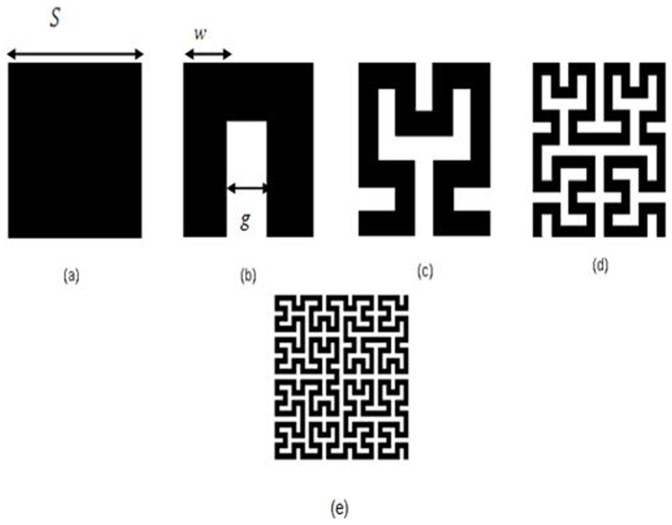
Hilbert fractal iterations (a) Original, 0^th^ iteration (b) 1^st^ iteration (c) 2^nd^ iteration (d) 3^rd^ iteration.



Hilbert fractal geometry represents the space-filling curves (SFCs). The composition of this shape can be prepared from a long conductive strip compacted within a microstrip patch as in Fig. 1. As the iteration of the curve increases, Hilbert fractal curve may space-fill the patch. It has been used in a wide variety of small antenna and filter designs [8], [11]. The fractal curve can be suitable in a square section of *S* as external side. For a Hilbert resonator, constructed from a thin metallic strip in the form of Hilbert curve with side dimension *S* and iteration *k*, the overall line segments *L*(*k*) can be calculated from [8]:

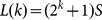
(1)The general aim of designing antennas and filters using Hilbert fractal geometry is to increase the iteration of the fractal curve as far as possible so as to match the resonator in more miniaturized area. On the other hand, it has been found during the use of Hilbert microstrip resonators, there is a tradeoff between miniaturization and quality factor of the resonator. For a microstrip resonator, the strip width *w* and the spacing between the strips *g* are the parameters which relatively define this tradeoff [8]. Both *w* and *g* are connected with the external side *S* and iteration level *k* (*k*≥2) by [8]:
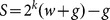
(2)


It can be concluded from (2) that higher levels of fractal iteration imply a lower value of microstrip width, consequently increasing the dissipative losses that will lead to analogous degradation of the quality factor [8].

## Filter Configurations and Simulation Results

**Figure 2:**
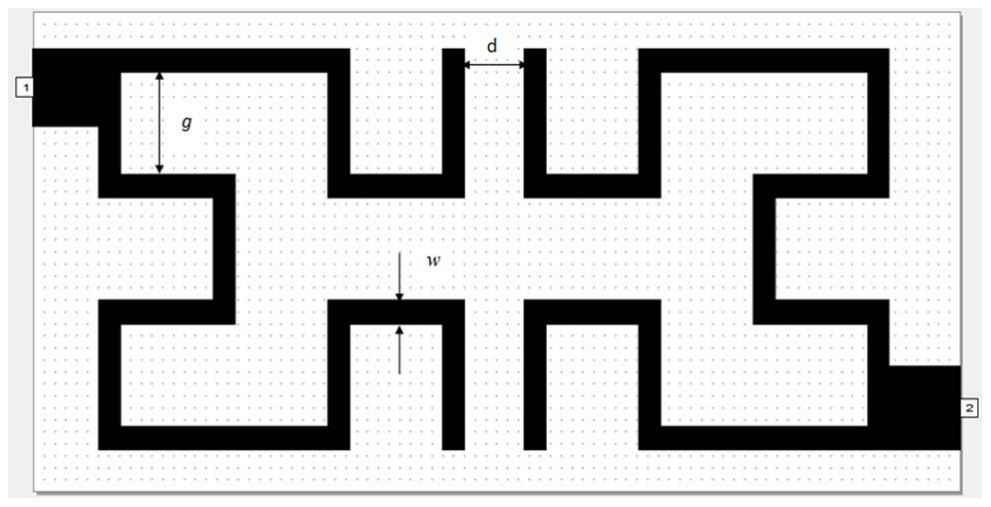
The modeled layout of WBPF.

In this study, the design of microstrip Hilbert fractal based filters has been realized by placing two resonators next to each other in specified distance. Each resonator is a physical component that stores both magnetic and electric energy in a frequency-dependent way. At fundamental frequency, the magnetic and electric current distributions in the resonator are equally stored. Hilbert fractal based resonators are well popular in planar filter applications, for they have more compact sizes, reasonable losses, better power handling features and more miniaturization as compared with meander structure or split ring resonators [4], [5], [8]. Accordingly, WBPF consists of two microstrip resonators as in Fig. 2, each resonator is based on 2^nd^ iteration of Hilbert fractal geometry. By the way, Hilbert resonator represents a single pole resonant circuit. So, the resulting two resonator bandpass filter will have two poles (2^nd^ order filter) regardless to the iteration number of the fractal geometry. It has been assumed that the proposed filter structure has been etched using RT/Duroid substrate 6010LM with a relative dielectric constant of 10.8, substrate thickness of 1.27 mm and metallization thickness of 35 *μ*m. Two 50 ohm feed lines as input and output (I/O) ports are placed in left up and right bottom corners of the filter. The width and length of these feeders are about 1.3 mm and 1.5 mm respectively. The proposed filter has overall dimensions of 13.5×6.7 mm^2^ with a trace width of about 0.4 mm, gap between strips of about 1.7 mm and edge spacing between the two resonators, d = 0.1 mm. The dimensions of this microstrip filter using electromagnetic modeling and simulation have been chosen by arbitrary trails and suitable scaling according to selected frequency of wireless communication systems. The layout of the proposed microstrip filter and dimension scaling method are essentially based on that presented in [18] and [19]. WBPF design has been simulated and evaluated using a full-wave based electromagnetic simulator Sonnet software package.

Sonnet EM simulator is based on the modified method of moment, such that it evaluates the filter response by dividing first the resonators in small divisions (mesh), less or more fitted according to the desired accuracy, and then solving a set of linear equations derived from an integral equation. The mesh division here has been chosen to be 1 mm. The filter has been run under frequency range from 1 GHz to 3.5 GHz with frequency step of 0.025 GHz. Suitable boundary conditions are assigned, and then meshing is carried out on the model to get final refined mesh. In meshing, it is well-known that a finer mesh (more divisions) will lead to a more precise solution. However, a finer mesh will also require more time for the computer to solve. Therefore, it is necessary to decide the proper balance between computation time and an acceptable level of accuracy. The stationary solver (including parametric sweeps) uses a linear solver algorithm for solution determination. The execution has been performed using Intel(R) Core(TM) i5-3770 @2.67 GHz CPU.

**Figure 3:**
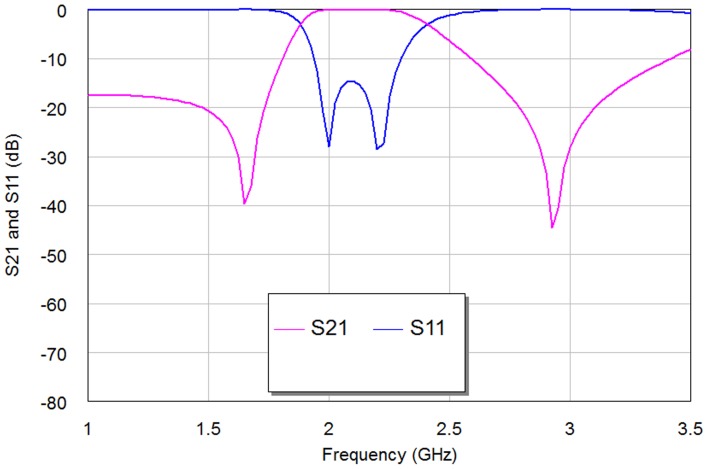
The return loss and transmission responses of WBPF.

**Figure 4:**
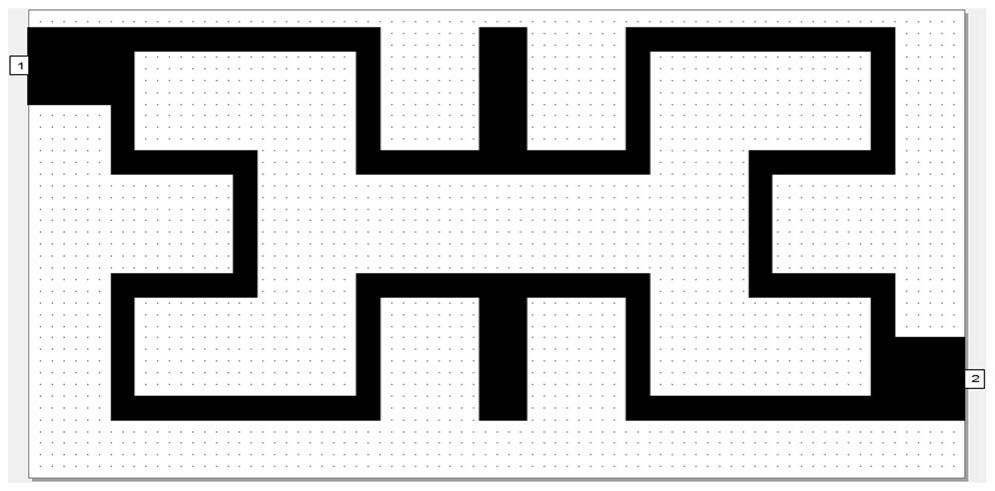
The modeled layout of NBSF.

**Figure 5:**
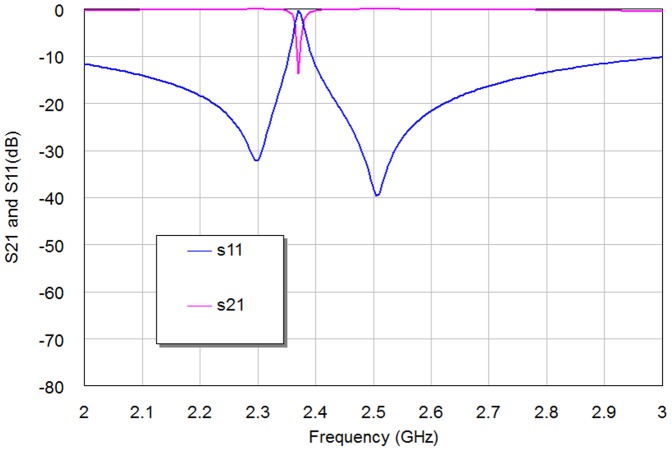
The return loss and transmission responses of NBSF.

The simulation results of return loss and transmission responses for WBPF are shown in Fig. 3. In this figure, the pass-band has two resonances at 2 and 2.2 GHz with a bandwidth of 0.52 GHz, −28 dB return loss and −0.125 dB insertion loss, can be observed clearly. The same resonators with depicted dimensions substrate specifications and simulator setting has been used to build NBSF, but with coupling edge spacing between the two resonators, d = 0 mm. The topology of this filter is shown in Fig. 4 with overall dimensions of 13.4×6.7 mm^2^. The filter is simulated under frequency range from 1 GHz to 3 GHz with frequency step of 0.025 GHz. The corresponding results of return loss and transmission responses are shown in Fig. 5. It has seen from this figure that the center frequency is 2.37 GHz and the rejection bandwidth is 20 MHz, while the return loss and insertion loss values are −0.1873 dB and 13.746 dB respectively. This NBSF can be used in broadband communication systems that are sensitive to fixed frequency interferences. It can be concluded from Figs. 3 and 5 that the simulation results of return loss, S11, and transmission, S21, responses of these filters offer good frequency responses with adequate performance. By the way, two transmission zeros (for WBPF) and reflection zeros (for NBSF) have been appeared in output frequency responses of proposed filters at finite frequencies near the pass-band and reject-band regions as depicted from Fig. 3 and Fig. 5 respectively. These responses are known as quasi-elliptic frequency responses for the designed filters. However, these responses and their consequent transmission and reflection zeros could be, to a certain extent, adjusted through the variation of edge coupling gap between Hilbert resonators and/or the input/output coupling used. For WBPF, two transmission zeros are located around resonant frequencies at 1.65 GHz and 2.9 GHz with S21 magnitudes of −39.73 dB and −44.742 dB, respectively while NBSF exhibits two reflection zeros of −32.176 dB and −38.227 dB at 2.3 GHz and 2.5 GHz, respectively.

In general, all passive resonating devices must have definite size in terms of the guided wavelength (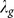) which can be calculated according to the following equations [6], [19]:
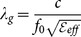
(3)

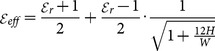
(4)where *c* is the velocity of light, 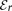 is the relative dielectric constant, 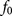 is the center frequency and 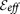 represents the effective dielectric constant that can be calculated from Eq. (4) and it depends obviously on the conductor width (*W*) and the substrate thickness (*H*). However, in the present work, effective dielectric constant has been approximated to 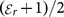. There are probably better approximations for this parameter; however the additional efforts to obtain more accurate 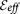 is still not worth it [20]. Based on above equations, the overall dimensions, in terms of 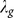, are found to be of (0.23 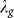
×0.11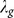) and (0.257 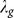
×0.13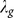) for WBPF and NBSF respectively. The degree of coupling depends on the values of the width (*w*) and gap (*g*) of Hilbert fractal curve strips, which also affects the resonant frequency of output response due to changes in *L* and *S* magnitudes according to Eqs. (1) and (2) [8], [11]. Consequently, the filter dimensions can be willingly varied upward or downward according to desired operating frequencies of wireless communication applications.

**Figure 6:**
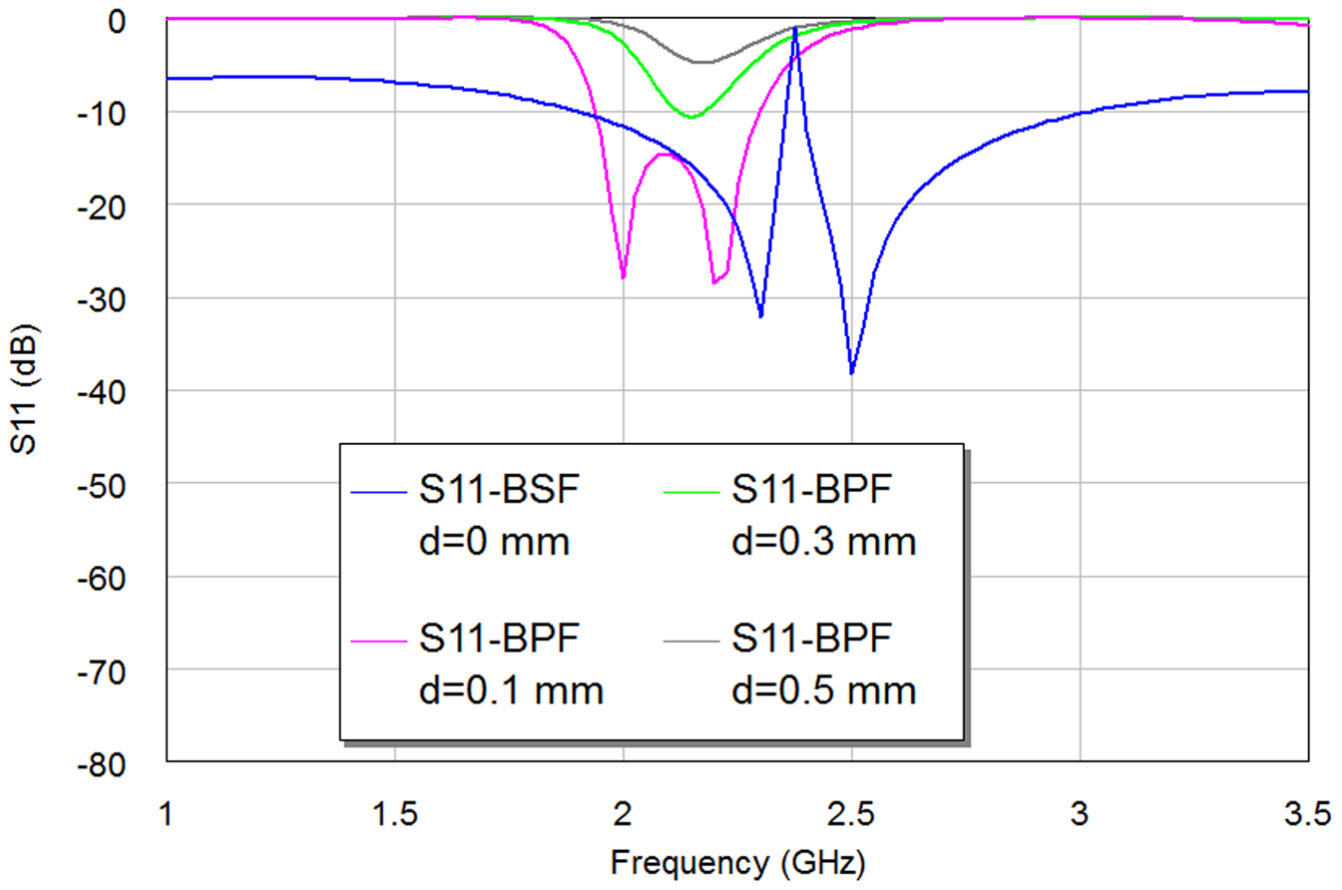
The transmission responses of the resulting 2^nd^ iteration Hilbert microstrip filter with respect to different edge spacing values, d, (in mm).

**Figure 7:**
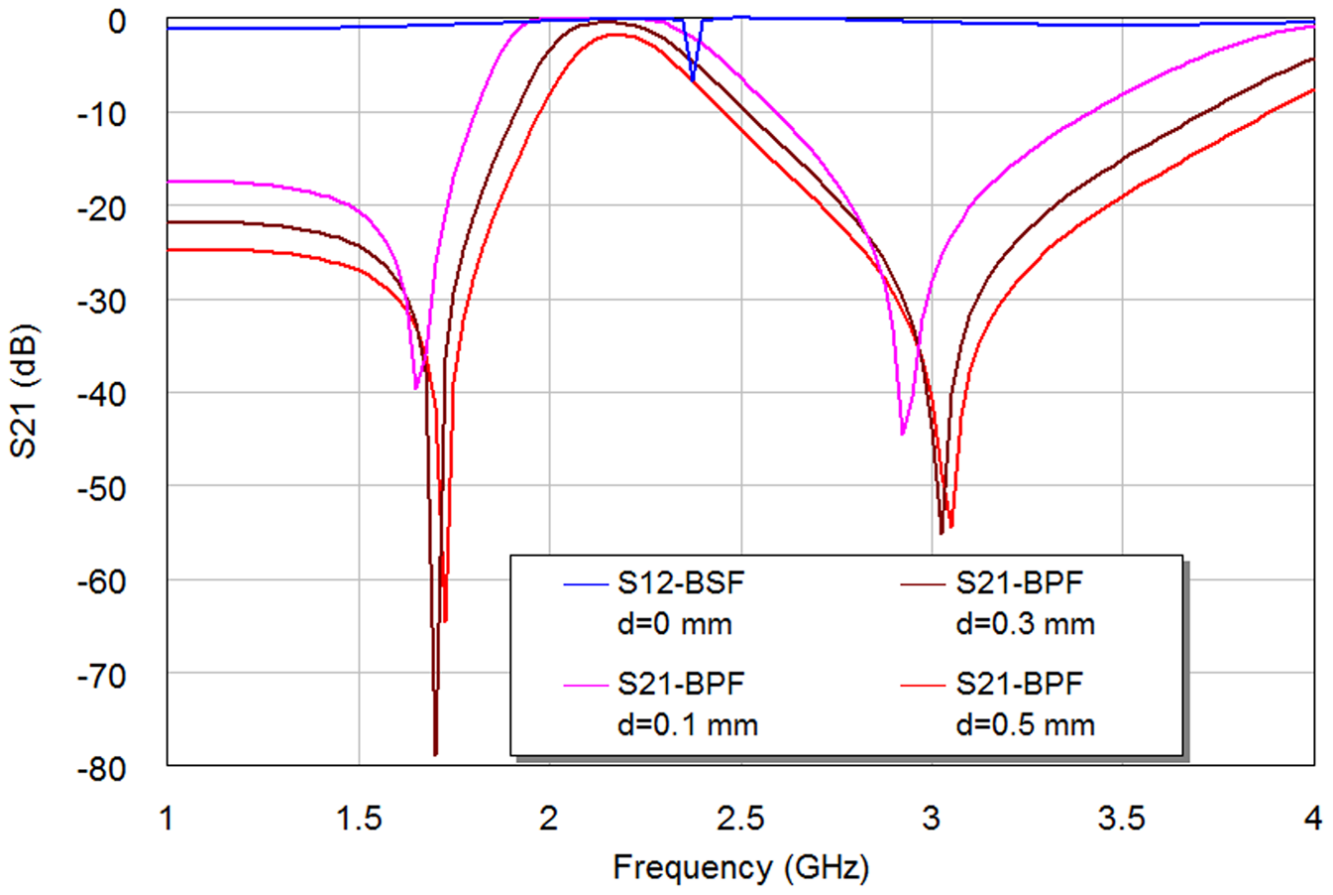
The return loss responses of the resulting 2^nd^ iteration Hilbert microstrip filter with respect to different edge spacing values, d, (in mm).

Besides the resonator dimensions, to reach to design frequency, there is also another vital parameter playing an important role in the resulting multi-resonator filter performance [11]. This is the spacing between the adjacent resonators (d). Its effect obviously appears in the return loss and insertion loss magnitudes more than on resonance. Moreover, this factor characterizes interaction of two resonators which is adopted mostly in resonator filter theory. This gap is also known as capacitive coupling and it couples these resonators electrically. On the other hand, the direct coupled resonators (at d = 0 mm) are interacted magnetically and, in other words, it represents inductive coupling. Parametric study to investigate the effects of this parameter on the resulting filter performance, will lead to minimum insertion loss and maximum return loss at the design frequency, as well as characterize the intended type of filter as pass or reject band. In this paper, we have used Hilbert microstrip resonators based on 2^nd^ iteration level as a clarification example for adopting the optimization process as it can be seen from Figs. 6–7 and Table 1. Figs. 6–7 show the resulting S11 and S21 responses corresponding to different values of the spacing between the two resonators for the 2^nd^ iteration of Hilbert fractal based filters. Table 1 shows the results of the modeled Hilbert filters with edge spacing as a parameter with d = 0 mm, 0.1 mm, 0.3 mm and 0.5 mm. It is clear, in both figures and Table 1; the variation in the spacing slightly affects the resonant frequency, while its effect is more noticeable on the transmission zeroes, return loss, insertion loss, bandwidth as well as the class of filter.

**Table 1:** Summary of simulation result parameters of the modeled Hilbert Filters with respect to d values.

	d = 0 mm (BSF)	d = 0.1 mm (BPF)	d = 0.3 mm (BPF)	d = 0.5 mm (BPF)
**Resonance Frequencies, GHz**	2.37	2,2.2	2.1384	2.1655
**Return Loss (dB)**	−0.1873	−28	−10.57	−4.812
**Insertion Loss(dB)**	−13.746	−0.125	−0.4	−2.158
**Actual Bandwidth (at −3 dB)**	20 MHz	520 MHz	331 MHz	170 MHz
**Trans. or Reflect. Zeros(dB)** *	−32.176, −38.227	−39.73, −44.742	−78.895, −55.157	−64.604, −54.414

*Trans. or Reflect. Zeros  =  Transmission or Reflection Zeros (The transmission zeros here have been predicted for BPF cases while reflection zeros have been predicted for BSF case).

**Figure 8:**
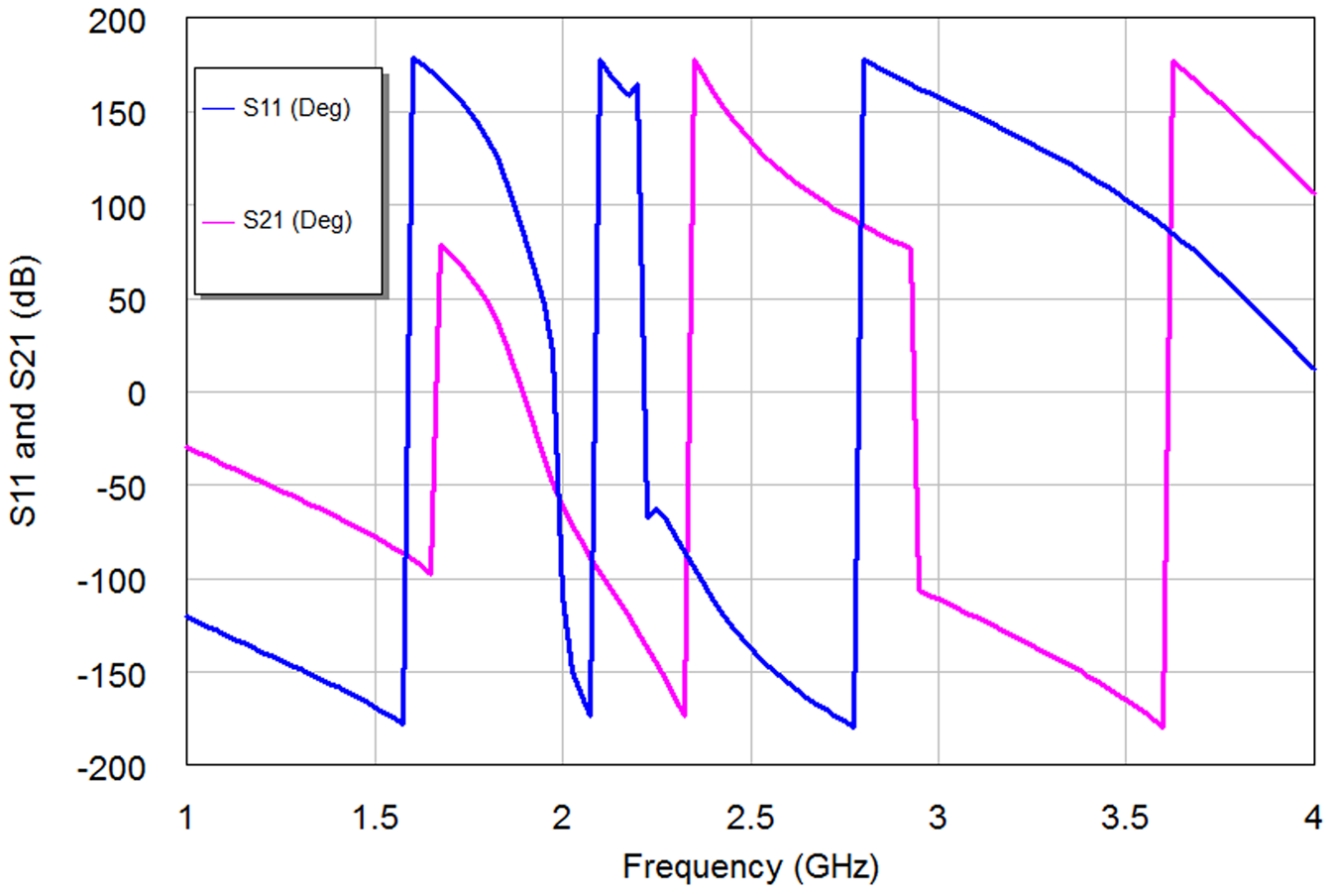
The phase responses of the resulting 2^nd^ iteration Hilbert microstrip WBPF.

**Figure 9:**
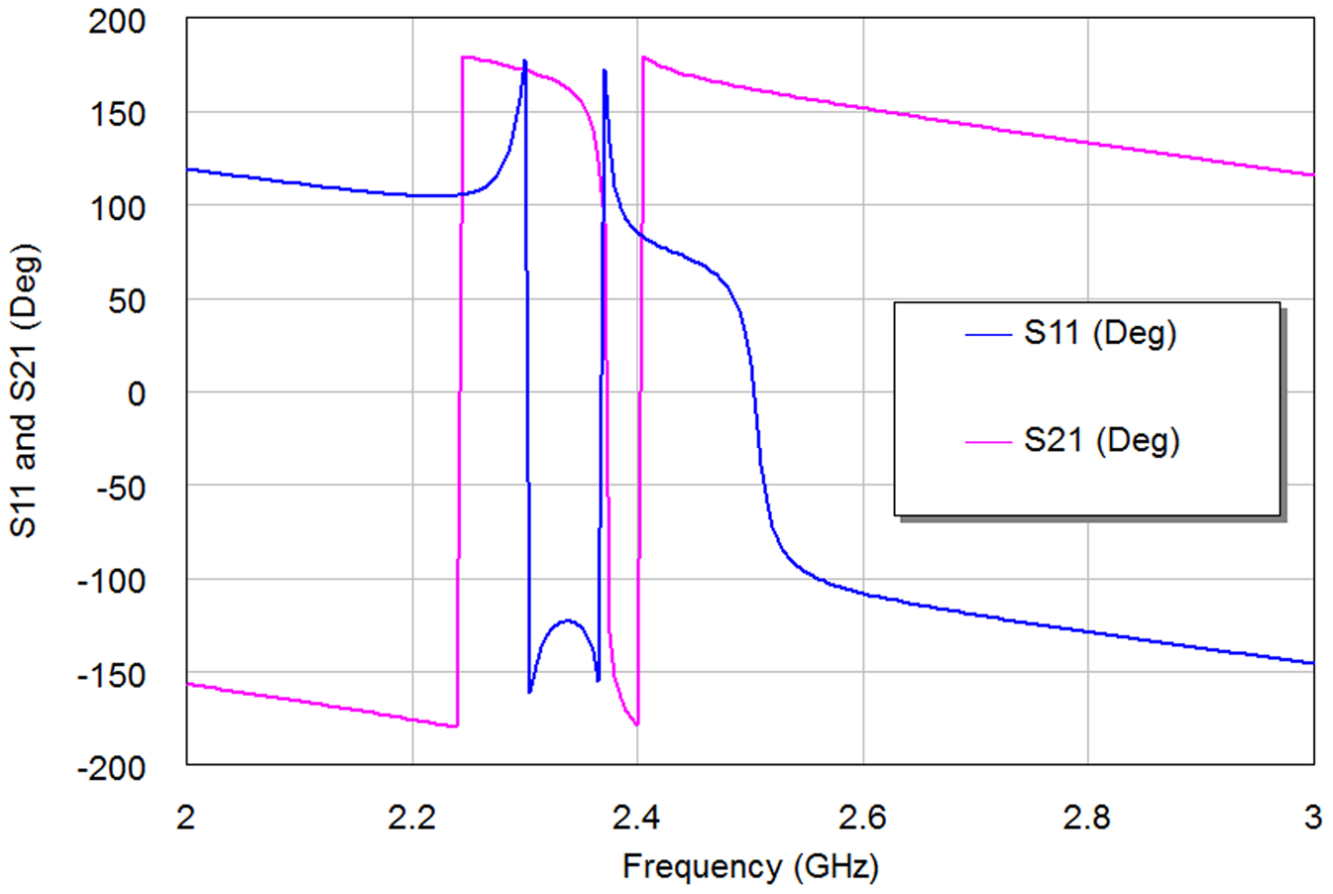
The phase responses of the resulting 2^nd^ iteration Hilbert microstrip NBSF.

The BSF response can be obtained with d = 0 mm as compared to BPF responses with other d cases. This is because of increased inductance of the filter structure without coupling gap case, consequently producing BSF response. Also, the simulation results involve that the gap spacing affects the position of the transmission zero on the right side of the passband slightly more than that of the left of the passband as in edge spacing values, 0.1 mm, 0.3 mm and 0.5 mm. The optimal responses of S11 and S21 for WBPF can be found in d = 0.1 mm case. Figs. 8–9 show the phase scattering parameters for S11 and S12 responses within the swept frequency range from 1 to 4 GHz and within output phase angle range from −200 to 200 degrees. These responses include some frequency jumps which are the significant properties of quasi-elliptic filters. Accordingly, the intersection between S11 and S21 responses can be recognized easily, especially nearby resonant frequency. However, the S11 scattering response for NBSF configuration offers lowest jumping rate than other scattering responses of proposed filters where obvious phase decay can be identified easily, especially after 2.37 GHz center frequency.

**Figure 10:**
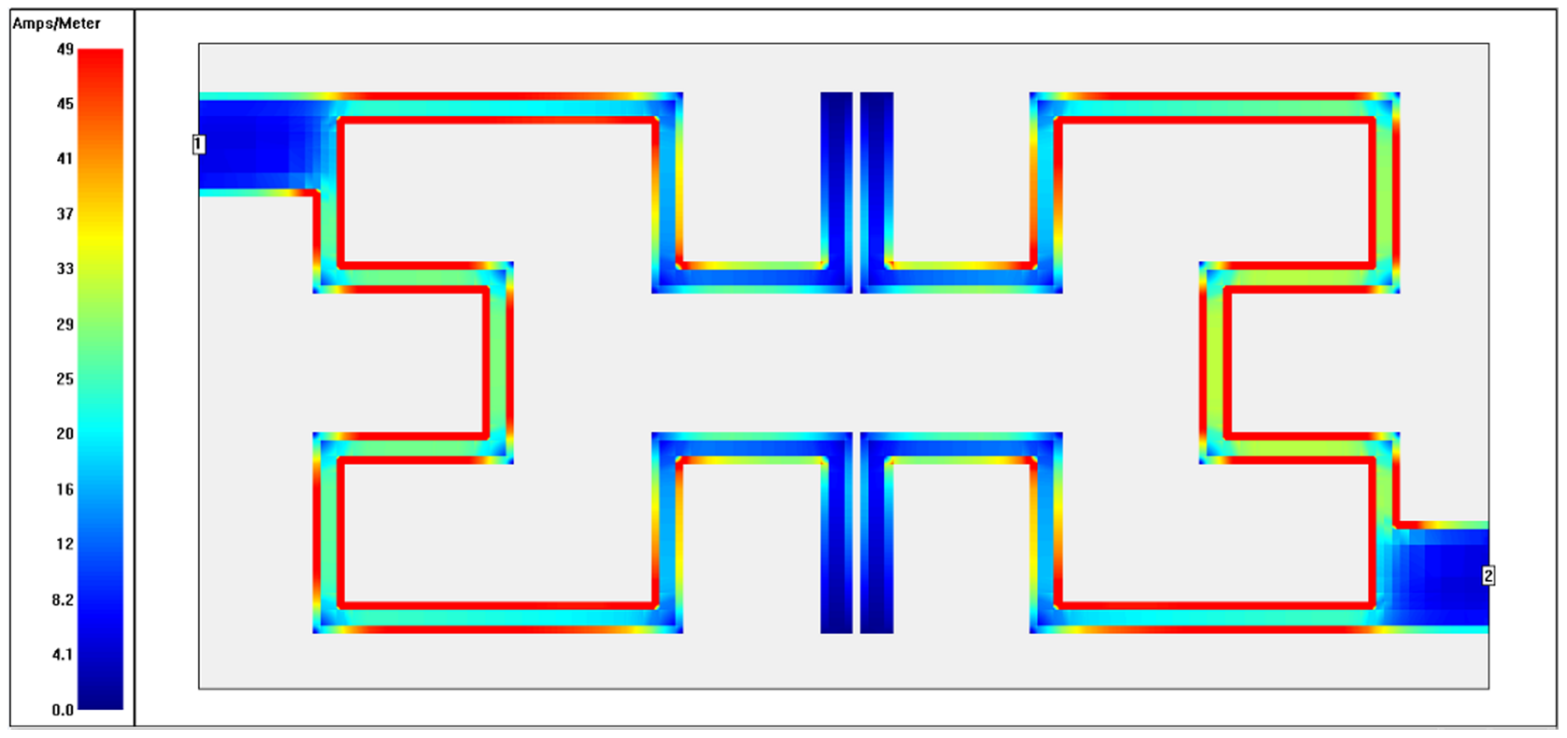
Current density distribution at the conducting surface of the 2^nd^ iteration Hilbert WBPF simulated at an operating frequency of 2 GHz.

**Figure 11:**
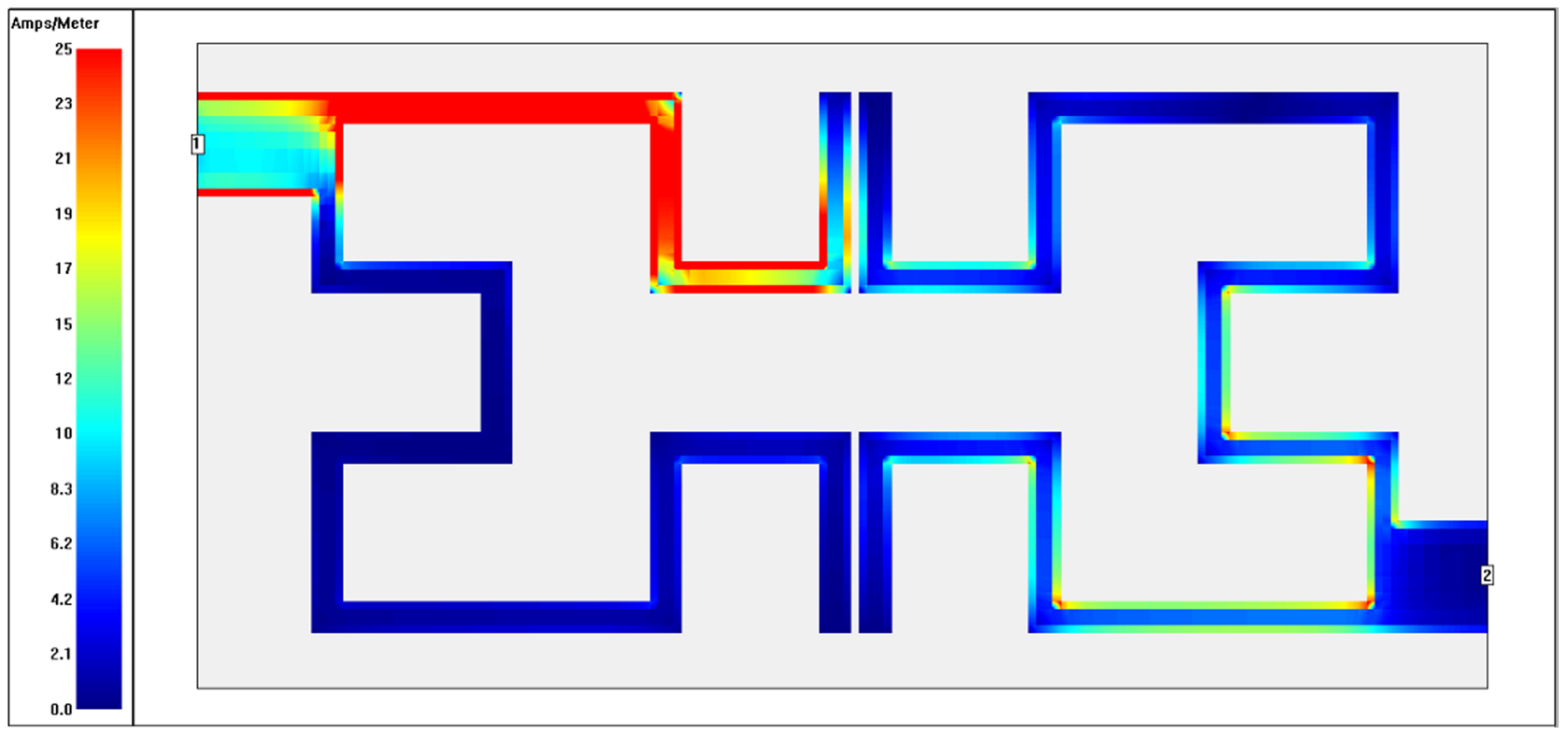
Current density distribution at the conducting surface of the 2^nd^ iteration Hilbert WBPF simulated at an operating frequency of 3 GHz.

**Figure 12:**
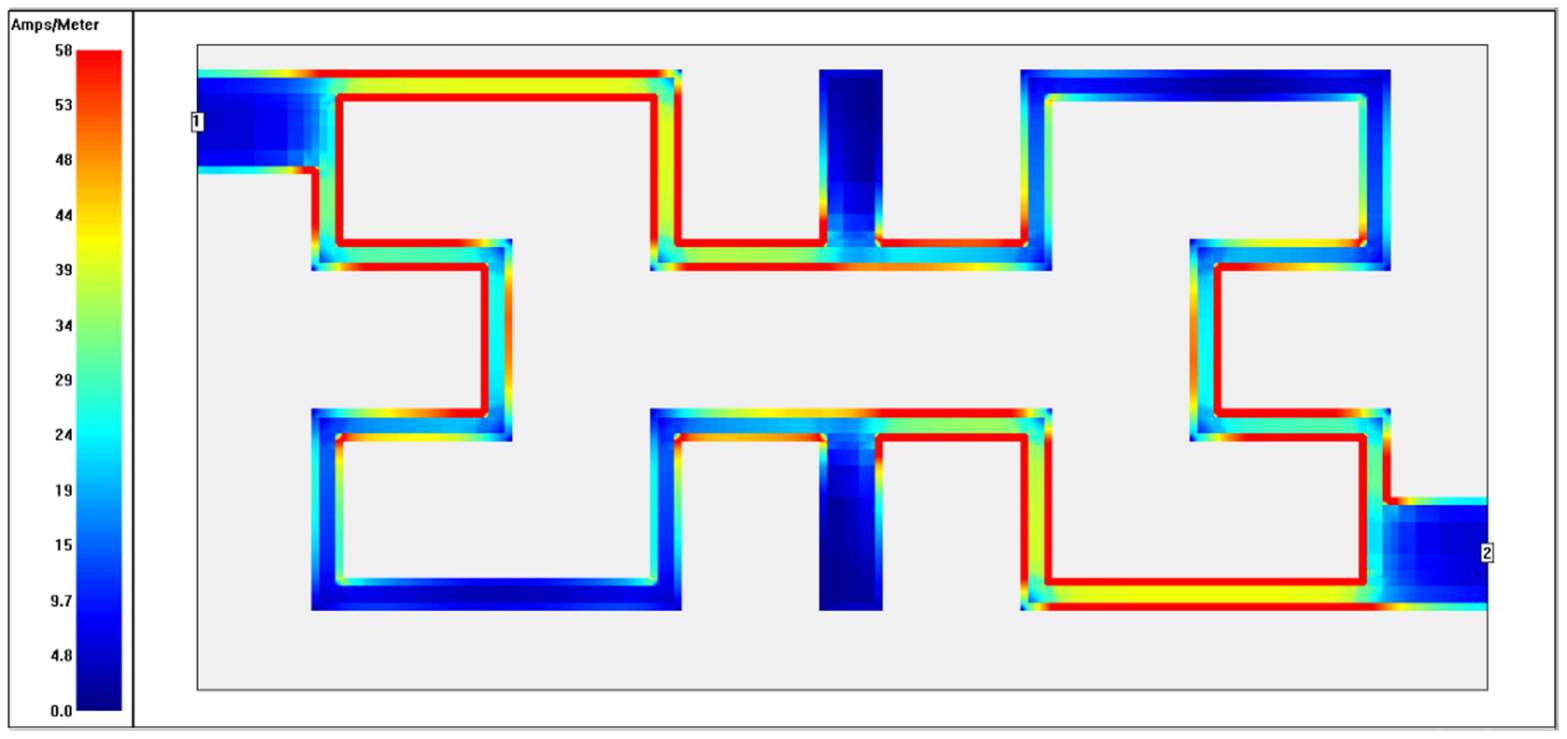
Current density distribution at the conducting surface of the 2^nd^ iteration Hilbert NBSF simulated at an operating frequency of 2.4 GHz.

**Figure 13:**
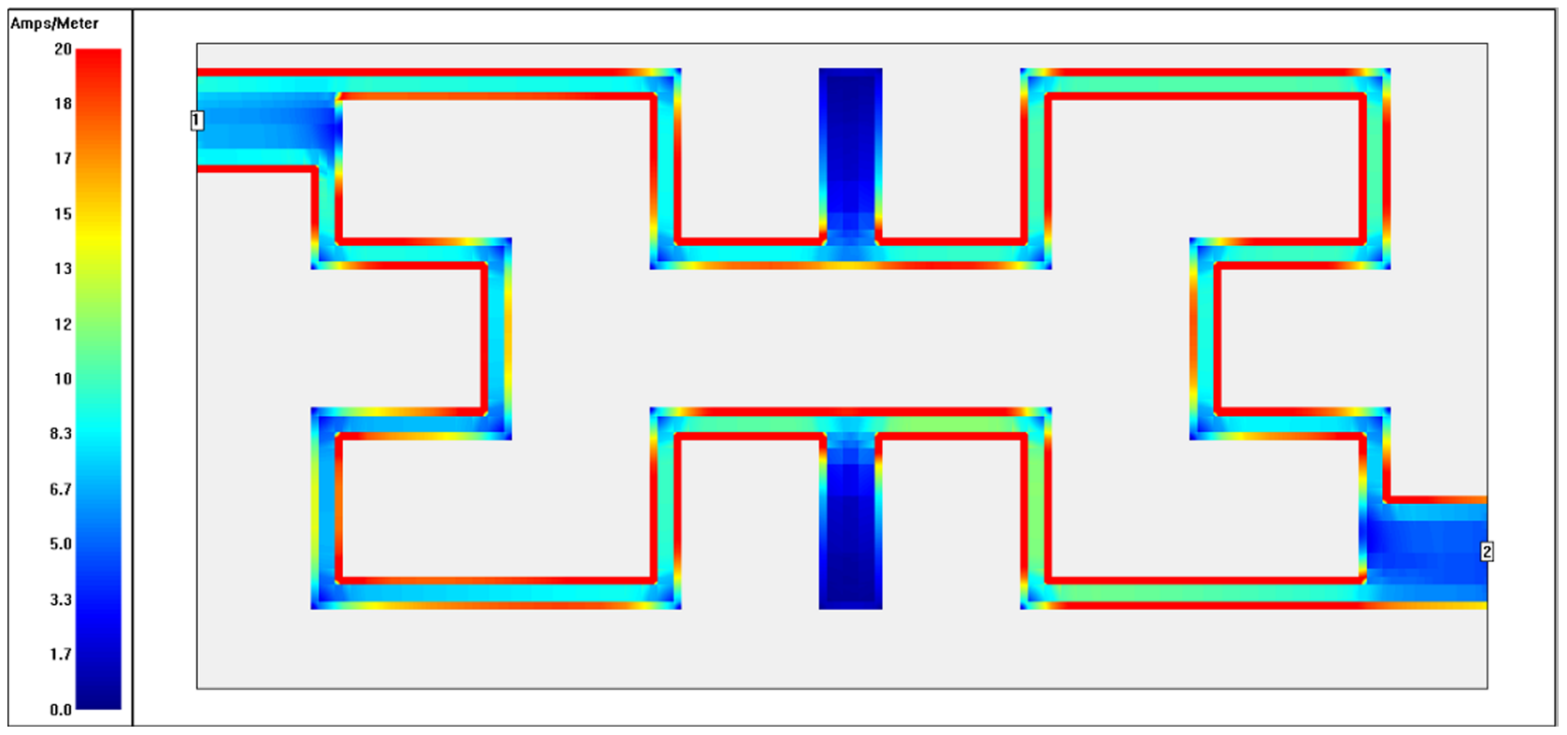
Current density distribution at the conducting surface of the 2^nd^ iteration Hilbert NBSF simulated at an operating frequency of 3 GHz.

To recognize which part of filter is being utilized (highest and lowest coupling regions) at each operating frequency, the surface current distributions are presented as in Figs. 10–13. These plots show surface current intensity graphs obtained by Sonnet simulator on the conducting surface of both Hilbert resonators. The surface current distributions are scaling themselves as second iteration Hilbert fractal geometry for each resonator. It is very clear from these figures that the current distributions differ from frequency to another where the red color indicates maximum coupling effect while blue color indicate the least one. The maximum surface current densities can be seen at the resonant frequencies for both WBPF and NBSF structures, which is due to the fact that the quasi-static resonance is being fully excited. Whereas, the lowest current intensities has been observed at 3 GHz in the stop-band region for WBPF and in the pass-band region for NBSF at the same frequency. In this case, weakest coupling can be seen, which is given by the fact that the designed filter are not being excited at 3 GHz.

## Conclusions

New WBPF and NBSF designs are introduced as well compact two-pole filters. The proposed microstrip fractal based filters have been composed of two resonators based on 2^nd^ iteration of Hilbert fractal geometry using a substrate having a dielectric constant of 10.8 and a thickness of 1.27 mm. WBPF has been designed at resonant frequencies of 2 and 2.2 GHz with a bandwidth of 0.52 GHz in pass-band region, while NBSF has a center frequency of 2.37 GHz with 20 MHz bandwidth in the stop-band region. It has been found the coupling edge spacing (d) affects the filter performances obviously, in addition to circuit type as pass or reject band. The proposed designs offer high performance and simple fabrication for the implementation of fractal microstrip filters, which can be modified to be suitable for a wide variety of communication systems.
